# A multi-faceted exploration of unmet needs in the continuing improvement and development of fertility care amidst a pandemic

**DOI:** 10.1590/S1677-5538.IBJU.2024.9915

**Published:** 2024-07-01

**Authors:** Monica Augustyniak, Giovanni Coticchio, Sandro C. Esteves, Markus S. Kupka, Chen Hong, Anita Fincham, Patrice Lazure, Sophie Péloquin

**Affiliations:** 1 AXDEV Group Inc. Brossard QC Canada AXDEV Group Inc., Brossard, QC, Canada; 2 IVIRMA Global Research Alliance Bologna Italy IVIRMA Global Research Alliance, 9.baby, Bologna, Italy; 3 ANDROFERT Clínica de Andrologia e Reprodução Humana Campinas SP Brasil ANDROFERT, Clínica de Andrologia e Reprodução Humana, Campinas, SP, Brasil; 4 Universidade Estadual de Campinas Divisão de Urologia Departamento de Cirurgia Campinas SP Brasil Departamento de Cirurgia, Divisão de Urologia, Universidade Estadual de Campinas (UNICAMP), Campinas, SP, Brasil; 5 Aarhus University Faculty of Health Aarhus Denmark Faculty of Health, Aarhus University, Aarhus, Denmark; 6 Ludwig-Maximilians -University Munich Germany Ludwig-Maximilians -University, Munich, Germany; 7 Shanghai First Maternity and Infant Hospital Shanghai China Shanghai First Maternity and Infant Hospital, Shanghai, China; 8 Fertility Europe Brussels Belgium Fertility Europe, Brussels, Belgium

**Keywords:** Infertility, Male, Needs Assessment, Pandemics, Andrology

## Abstract

**Purpose:**

The continuous improvement and development of fertility care, internationally, requires ongoing monitoring of current delivery processes and outcomes in clinical practice. This descriptive and exploratory mixed-methods study was conducted in eight countries (Brazil, China, France, Germany, Italy, Mexico, Spain and the United Kingdom) to assess the unmet needs of fertility patients (male and female), and existing challenges, barriers and educational gaps of physicians and laboratory specialists involved in human fertility care during the COVID-19 pandemic.

**Materials and Methods:**

The study was deployed sequentially in two phases: 1) in-depth 45-minute semi-structured interviews (n=76), transcribed, coded and thematically analysed using an inductive reasoning approach, 2) an online survey (n=303) informed by the findings of the qualitative interviews, face validated by experts in reproductive medicine, and analysed using descriptive and inferential statistical methods.

**Results:**

The integrated results of both phases indicated numerous areas of challenges, including: 1) investigating male-related infertility; 2) deciding appropriate treatment for men and selective use of assisted reproductive technology; and 3) maintaining access to high-quality fertility care during a pandemic.

**Conclusions:**

The paper presents a reflective piece on knowledge and skills that warrant ongoing monitoring and improvement amongst reproductive medicine healthcare professionals amidst future pandemics and unanticipated health system disruptions. Moreover, these findings suggest that there is an additional need to better understand the required changes in policies and organizational processes that would facilitate access to andrology services for male infertility and specialized care, as needed.

## INTRODUCTION

It may be worthwhile to revisit a recent event that substantially disrupted health systems and reproductive medicine at a global scale: the COVID-19 pandemic. The first seven cases of hospitalized pneumonia due to the severe acute respiratory syndrome coronavirus 2 (SARS-CoV-2, also known as COVID-19) were reported and investigated in Wuhan Jin Yin-Tan Hospital, China, in December 2019 ( 1 ). Shortly after, in March 2020, the World Health Organization (WHO) announced a state of pandemic ( 2 ). Outbreak measures were recommended by professional health societies and installed by national public health authorities including suspension of healthcare services considered "non-urgent". Fertility care fell under this categorization.

Professional associations in reproductive medicine recommended discontinuation of fertility care services, particularly the use of assisted human reproductive technology (ART) and andrological evaluation, for new patients and those without cancer diagnoses ( 3 , 4 ). This recommendation was informed by the lack of evidence pertaining to the effect of COVID-19 infection on pregnancy and neonatal outcomes, and the need to ensure physical distancing as preventative and precautionary measures. The impact of this recommendation was quickly felt.

In 2020, a survey administered to 207 individual fertility care centres across 97 countries showed that 83% of the respondents reported no or limited access to ART treatments in their country, and 40% reported changes in policies regarding fertility treatments offered to their patient population ( 5 ). Patients’ psychological distress associated with a drastic lack of services, especially for those with a narrow window for successful conception via ART, was reported ( 6 – 8 ).

A call to reconsider and adapt policies surrounding access to ART was made to ensure reproductive care was not "unfairly curtailed" to low-prognosis patients ( 9 – 13 ). These urgent requirements for change were only additions to previously identified challenges in the field: ensuring evaluation of male-related factors prior to selecting an ART procedure, such as intracytoplasmic sperm injection (ICSI) ( 14 , 15 ); reaching consensus on the appropriate stage of embryo cryopreservation or vitrification ( 16 ); using ART in alignment with best-practice guidelines ( 17 ); providing effective psychological support for patients ( 18 ); and tailoring communication to meet patient needs ( 17 ).

Amidst all these changes and growing needs, healthcare professionals (HCPs) involved in reproductive medicine are also expected to stay abreast of, and integrate continuing advances in their practice as needed, including: integrating updated recommendations and guidelines ( 10 , 19 – 22 ), emerging assessments tools ( 23 , 24 ), and newly available treatments, such as those addressing aetiologies of male infertility ( 25 ).

With the aim of supporting reproductive medicine HCPs in effectively meeting their professional expectations during and beyond the COVID-19 pandemic, via evidence-based continuing medical education (CME), professional development (CPD) activities and other types of health system interventions, this study assessed priority needs for improvement in fertility care from the perspective of patients (male and female), and remaining challenges, barriers and educational gaps affecting physicians and laboratory specialists involved in ART and the treatment of male and/or heterosexual couples’ infertility.

## MATERIALS AND METHODS

An exploratory sequential mixed-methods study was conducted, involving a triangulation of data sources, methods, and interpretation viewpoints ( 26 , 27 ). This approach was selected to obtain a fulsome capture of the examined phenomena, in line with the study objectives, superior to what could be obtained with quantitative or qualitative approaches alone ( 28 ). An equal priority was given to both methods. The first phase (June 2021-August 2021) involved qualitative interviews with HCPs (physicians and laboratory specialists involved in ART and male infertility) and patients (male and female), to explore the context and meaning of challenges experienced in fertility care. The second phase (November 2021) used a quantitative survey informed by the findings of the qualitative interviews to measure the frequency and magnitude of gaps and challenges, in a distinct and larger sample of HCPs. The final phase involved integrating all findings to identify converging themes ( 26 , 29 ), and underlying educational gaps. Gaps were defined as the discrepancy between current and ideal states of knowledge, skills, beliefs, and performance of HCPs ( 30 ). The study protocol was reviewed and approved by Veritas Independent Review Board Inc. in accordance with ethical guidelines and regulations of the countries in which the study was conducted.

### Recruitment

E-mail invitations describing the study were sent to prospective participants in Brazil, China, France, Germany, Italy, Mexico, Spain, and the United Kingdom (UK). For each study phase, unique panels of HCPs and patients registered to receive invitations for healthcare research were used. Panels operated in compliance with the International Chamber of Commerce (ICC) and European Society for Opinion and Marketing Research (ESOMAR) guidelines. Invitations included a link to an online screening questionnaire and consent form. Phase 1 inclusion criteria were: 1) male or female patients, 18 to 55 years of age, actively seeking fertility care, and having attempted conception with ART at least once; or 2) actively practicing physicians specialised in reproductive medicine, reproductive endocrinology, obstetrics and/or gynecology (OBGYN) or andrology, with a minimum of five years in practice, involved in the diagnosis, treatment and/or management of infertility, and at least 500 ART-related procedures conducted over the last 12 months; or 3) actively practicing embryologists, andrology laboratory specialists, biologists, or microbiologists with a minimum of five years in practice, involved in the manipulation of human gametes or embryos of at least 10 patients per year for the purpose of ART. Phase 2 was conducted with HCPs only. Inclusion criteria were similar to phase 1, except: physicians could specialise in reproductive urology, all were required to provide care to male patients; a minimum of three years in practice experience and yearly caseload of at least 100 patients undergoing ART. All participants provided informed consent prior to enrolling. Purposive sampling was applied on an ongoing basis to ensure a variety of perspectives and profiles were obtained across samples ( 31 ).

### Phase 1: Qualitative Interviews (March – September 2021)

An interview guide was developed based on challenge areas identified in the literature ( Appendix-1 ). Questions were open-ended to elicit robust, descriptive responses, and allow for discussion of experiences and perspectives relevant to different professions and patients ( 32 ). Probes were used when explanations or contextualisation were needed. Final materials were translated into French, German, Italian, Mandarin, and Portuguese.

Semi-structured 45-minute interviews were conducted with trained moderators over a secure conference call in the participant's language, and recorded upon consent. Recordings were transcribed and imported into NVivo Version 12 software (QSR International Pty Ltd., 2021) for coding or organisation into a framework of relevant topics. If unanticipated but relevant content emerged from data analysis, a new code was created to integrate the topic into the analysis framework. Three researchers, including co-author MA coded the transcripts. Inter-coder reliability test results demonstrated fair consistency and reliability (Agreement rate > 90% amongst coders) ( 33 ). Data were thematically analysed using an inductive reasoning approach ( 34 ).

### Phase 2: Quantitative Survey (October – December 2021)

A 20-minute survey was developed by co-authors MA, PL and SP based on phase 1 findings. The HCP survey was face-validated by subject matter experts in reproductive medicine (co-authors GC, SE, MK, CH) and a patient organisation representative (co-author AF). The survey consisted of twenty-two closed-ended questions in the form of rating with five-point Likert-type scales (e.g., 1-no knowledge/skill, 2-basic, 3-intermediate, 4-advanced, 5-expert knowledge/skill) or multiple-choice response options, summing up to 174 survey items. Survey items were split between physicians caring exclusively for males; physicians caring for both sexes; and laboratory specialists and assessed knowledge, skill, beliefs (or attitudes), and performance in clinical practice ( 30 , 35 ). A clinical case question was included to help evaluate HCP decision-making when investigating and treating a couple's infertility.

The minimal targeted sample size (n=176) for the survey was calculated to reach a statistical power of 0.8 with α=0.05 and a large effect size (Cohen's w=0.5) for a 2x4 chi-square test ( 36 ), to account for comparison between four regions: South America, Western Europe, Southern Europe and Asia (n=44 per region). With the aim of strengthening descriptive comparisons by country, the final sample size was permitted to increase until survey closure. Survey responses were imported into SPSS Statistics (Version 27.0, IBM Corp., Armonk, NY, USA) for frequency and crosstabulation analysis with chi-square statistical tests. Appendix-2 presents full questions and responses discussed in the manuscript, as well as how each survey response was transformed for crosstabulation analysis.

### Data Integration and Trustworthiness

Integration of mixed methods was achieved by ensuring phase 1 findings informed the development of measures for phase 2 ( 27 ). In the reporting of findings, quotes representative of the identified challenge were further integrated by the co-authors to articulate meaning and context, while statistics from phase 2 were integrated to quantify the extent to which gaps and barriers were identified. The integration of mixed methods was first completed by researchers MA, PL and SP, and then reviewed in collaboration with co-authors CG, SCE, MK, CH and AF via online discussions.

## RESULTS

Seventy-six interviews and 303 surveys were completed ( Table-1 ). Phase 1 included female (n=23) and male (n=5) patients; the majority of who were over the age of 34 (F:70%; M:100%) and had received assisted conception, such as *in vitro* fertilization (IVF) (F:91%, M:80%) and medication, such as gonadotropins (F: 91%, M:60%). Phase 1 physicians were specialised in OBGYN (42%, 10/24), reproductive medicine (38%, 9/24) or endocrinology (21%, 5/24), while laboratory personnel included embryologists (70%,16/23), biologists (17%, 4/23), laboratory managers (9%, 2/23) and microbiologists (4%, 1/23). Phase 2 physicians were specialised in reproductive medicine (55%, 124/224), endocrinology (24%, 53/224), OBGYN (29%, 66/224), reproductive urology (17%, 39/224) and andrology (15%, 15/224), while laboratory personnel included embryologists (52%, 51/99), laboratory specialists (43%, 43/99) and biologists (5%, 5/99). The median number of ART-related procedures conducted by HCPs was 550 per year in phase 1, and 300 per year in phase 2.

**Table 1:** Sample Demographics.

Demographics		Phase 1 (Qualitative Interviews) n=76			Phase 2 (Quantitative Survey) n=323	
		PHYS a (n=24)	LAB b (n=24)	PX c (n=28)	PHYS a (n=224)	LAB b (n=99)
**Region**	**South America**	6 (26%)	6 (26%)	8 (29%)	54 (24%)	24 (24%)
	**Western Europe**	9 (38%)	9 (38%)	9 (32%)	75 (34%)	36 (36%)
	**Southern Europe**	6 (25%)	6 (25%)	8 (29%)	54 (24%)	24 (24%)
	**Asia**	3 (13%)	3 (13%)	3 (11%)	40 (18%)	15 (15%)
**Country**	**Brazil**	3 (13%)	3 (13%)	4 (14%)	27 (12%)	12 (12%)
	**China**	3 (13%)	3 (13%)	3 (11%)	40 (18%)	15 (12%)
	**France**	3 (13%)	3 (13%)	3 (11%)	26 (12%)	12 (12%)
	**Germany**	3 (13%)	3 (13%)	3 (11%)	25 (11%)	12 (12%)
	**Italy**	3 (13%)	3 (13%)	4 (14%)	27 (12%)	12 (12%)
	**Mexico**	3 (13%)	3 (13%)	4 (14%)	27 (12%)	12 (12%)
	**Spain**	3 (13%)	3 (13%)	4 (14%)	27 (12%)	12 (12%)
	**UK**	3 (13%)	3 (13%)	3 (11%)	25 (11%)	12 (12%)
**Setting**	**Community standalone** **	7 (29%)	9 (38%)	-	91 (41%)	55 (56%)
	**Community hospital**	7 (29%)	8 (33%)	-	54 (24%)	37 (37%)
	**Academic hospital**	10 (42%)	7 (29%)	-	79 (35%)	7 (7%)
**Years of practice**	**3-10 years** *	5 (21%)	8 (33%)	-	81 (36%)	51 (52%)
	**11-20 years**	14 (58%)	12 (50%)	-	110 (49%)	42 (42%)
	**> 20 years**	5 (21%)	4 (17%)	-	33 (15%)	6 (6%)
**Age**	**25-34 years**	-	-	7 (25%)	-	-
	**35-44 years**	-	-	16 (57%)	-	-
	**> 44 years**	-	-	5 (18%)	-	-
**Sex**	**Female**	15 (63%)	11 (46%)	23 (82%)	97 (43%)	60 (61%)
	**Male**	9 (38%)	13 (54%)	5 (18%)	124 (55%)	37 (37%)

a PHYS: Reproductive medicine specialists, endocrinologists, obstetricians and gynecologists, reproductive urologists or andrologists involved in ART;

b LAB: Microbiologists, biologists or embryologists involved in the manipulation of gametes or embryos for the purpose of ART;

c PX: Male and female patients of fertility age, with full reproductive organs, seeking care due to difficulty conceiving and have attempted at least once ART.

* Minimum years of practice for phase 1 was 5 years, compared to 3 years for phase 2.

** Standalone community practice settings included: single-specialty or solo practice in andrology, gynecology or fertility care, as well as multi-speciality clinics or centres outside of hospitals.

**Figure 1:**
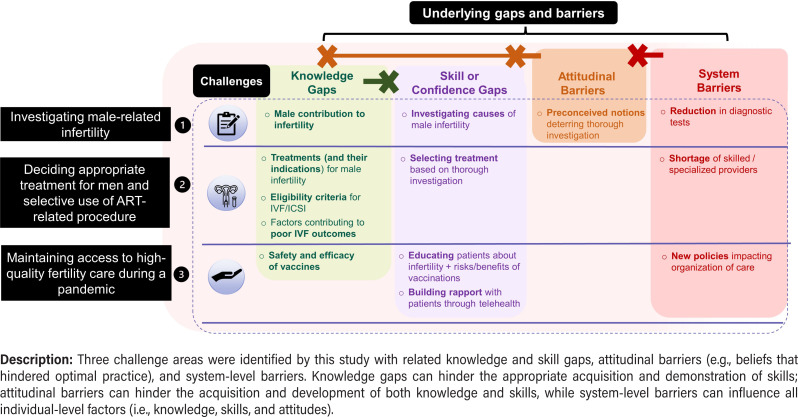
Three challenge areas in fertility care with underlying gaps and barriers.

The following themes and areas of challenges for HCPs emerged from the integration of results from both phases: 1) investigating male-related infertility; 2) deciding appropriate treatment for men with selective use of ART-related procedure; and 3) maintaining access to high-quality fertility care during a pandemic. Figure-1 outlines each area, underlying gaps and barriers hindering optimal care.

### 1) Challenges Investigating Male-Related Infertility

A knowledge gap of the extent of male contribution to couples’ infertility was found, in addition to skill gaps and preconceived notions deterring a thorough investigation of male infertility. Over a third (38%, 123/323) of surveyed HCPs responded that males contributed to 30% or less of all cases of couples’ infertility. A greater proportion of HCPs from China (58%, 32/55), Mexico (69%, 27/39) and Germany (43%, 16/37) demonstrated this knowledge gap, compared to HCPs from other countries ( Table-2 ). On average, 31% (100/318) of physicians rated their skill level as less than advanced or expert (i.e., as none, basic or intermediate) when investigating male causes of infertility. The percentage of participants reporting suboptimal skill levels was greatest amongst physicians specialised in reproductive medicine, endocrinology or OBGYN (38%, 65/169) than reproductive urology or andrology (11%, 6/53). Patient interviews indicated HCPs lack consideration of the factors, other than female age, that might contribute to difficulty conceiving:

*It was my wife who wanted to get pregnant. Thus, I let her organise everything. All was focused on her. All the tests that were done were focused on her. When we visited the last doctor, he focused on me instead. He explained that it was me who was unable to have children.*

– Male patient (45 years of age), Mexico

**Table 2:** – Percent of physicians with gaps in knowledge (K) and skill (S) by country.

Gap	Area of care	Countries (%, n)								
		South America		Western Europe			Southern Europe		Asia	Total Mean
		BR	MX	DE	FR	UK	IT	SP	CH	
K	Male contribution to infertility *	18% (7/39)	69% (27/39)	43% (16/37)	26% (10/38)	29% (11/37)	26% (10/39)	26% (10/39)	58% (32/55)	38% (123/323)
K	Eligibility for IVF and ICSI *	11% (3/27)	15% (4/27)	20% (5/25)	8% (2/26)	8% (2/24)	4% (1/26)	11% (2/27)	38% (15/39)	16% (35/221)
K	Microsurgical reconstruction of the male genital tract *	76% (19/25)	69% (18/26)	40% (10/25)	38% (10/26)	63% (15/24)	68% (18/27)	68% (18/27)	66% (25/38)	61% (133/218)
K	Transurethral resection of ejaculatory ducts *	88% (21/24)	70% (19/27)	24% (6/25)	44% (11/25)	63% (15/24)	52% (14/27)	68% (18/27)	58% (22/38)	58% (126/217)
K	Techniques for varicocele repair *	72% (18/25)	59% (16/27)	24% (6/25)	23% (6/26)	50% (12/24)	48% (13/27)	48% (13/27)	55% (21/38)	48% (105/219)
K	Empirical medical treatment with selective estrogen receptor modulators, antioxidants, and gonadotropin in males	65% (17/26)	33% (9/27)	47% (18/38)	48% (12/25)	54% (13/24)	41% (11/27)	56% (15/27)	47% (18/38)	47% (102/219)
K	Gonadotropin therapy for hormonal disorders in males *	74% (20/27)	26% (7/27)	33% (13/39)	23% (6/26)	26% (6/23)	41% (11/27)	44% (12/27)	33% (13/39)	36% (80/220)
K	Treatments for male patients that can improve IVF and ICSI outcomes *	31% (8/26)	30% (8/27)	16% (4/25)	27% (7/26)	25% (6/24)	19% (5/27)	33% (9/25)	50% (19/38)	30% (66/220)
K	Effectiveness of vaccines in preventing SARS-CoV-2 infections and limiting symptom severity	44% (15/34)	72% (28/39)	40% (14/35)	26% (10/38)	57% (21/37)	47% (17/36)	42% (15/36)	56% (29/52)	49% (149/307)
S	Investigating hypothalamic-pituitary axis dysfunction in males *	44% (12/27)	30% (8/27)	12% (3/25)	23% (6/26)	28% (7/25)	25% (6/24)	38% (10/26)	56% (22/39)	34% (74/219)
S	Investigating spermatogenic defects	42% (16/38)	18% (7/38)	24% (9/37)	34% (13/38)	36% 13/36)	32% (12/38)	44% (17/39)	48% (26/54)	36% (113/318)
S	Investigating ductal obstruction or dysfunction in males	44% (12/27)	44% (12/27)	24% (6/25)	32% (8/25)	44% (11/25)	32% (8/25)	44% (12/27)	41% (16/39)	39% (85/220)
S	Investigating infectious disease causes of infertility in males	33% (9/27)	19% (5/27)	16% (4/25)	23% (6/26)	52% (13/25)	35% (9/26)	52% (14/27)	44% (17/39)	35% (77/222)
S	Investigating systemic causes of infertility in males	37% (10/27)	18% (5/27)	20% (5/25)	19% (5/26)	17% (4/24)	19% (5/26)	41% (11/27)	41% (16/39)	28% (61/221)
S	Counselling patients on the safety and efficacy of available vaccines (including ones for SARS-CoV-2) *	11% (3/27)	40% (10/25)	28% (7/25)	27% (7/26)	42% (10/24)	42% (10/24)	44% (12/27)	67% (26/39)	39% (85/217)
S	Discussing with patients’ evidence regarding the risks versus benefits of SARS-CoV-2 vaccines on pregnancy and birth outcomes *	15% (4/27)	41% (11/27)	20% (5/25)	31% (8/26)	40% (10/25)	48% (12/25)	41% (11/27)	67% (26/39)	40% (89/221)
S	Building rapport with patients via telehealth during an initial evaluation *	8% (2/25)	56% (14/25)	16% (4/25)	32% (8/25)	12% (3/25)	44% (11/25)	22% (4/27)	48% (19/40)	31% (67/217)

K (Knowledge gap); S (Skill gap); BR (Brazil); MX (Mexico); DE (Germany); FR (France); UK (United Kingdom); IT (Italy); SP (Spain); CH (China).

* Asymptotic significance (2-sided) < 0.05 for 2x4 (gap X region) crosstabulation with Pearson Chi-Square statistical test.

Of physicians involved in the care of both sexes, 32% (60/188) reported not always considering the health of male partners when investigating and treating infertility. An average of 43% (138/323) of surveyed HCPs agreed or strongly agreed with the statement "causes of male infertility are simpler to investigate than female infertility." The agreement was less prominent among physicians and laboratory specialists in Italy and Spain (29%, 23/78, Appendix-3 ).

### 2) Challenges in Deciding Appropriate Treatment for Men and Selective Use of ART-related procedures

Knowledge gaps regarding available treatments to address male infertility, and uncertainty about ART eligibility criteria were found. Procedures and treatments where the highest proportion of physicians rated their knowledge as less than advanced or expert were: microsurgical reconstruction of the male genital tract (61%, 133/218), transurethral resection of ejaculatory ducts (58%, 126/217), techniques for varicocele repair (48%, 105/219), and empirical medical treatment with selective estrogen receptor modulators, antioxidants and gonadotropin for males (47%, 102/219). When considering multiple potential treatments for male patients, 43% (70/162) of specialists in reproductive medicine, endocrinology, and OBGYN had a knowledge gap, compared to 10% (5/50) in reproductive urology and andrology (p<0.001).

A majority (85%, 275/323) of surveyed HCPs across all countries either were unsure of, or incorrectly agreed with the statement that "even very low rates of sperm morphology have poor predictive power" for IVF, and "poor embryonic development in an IVF cycle is an indication for ICSI", despite evidence demonstrating the contrary ( Appendix-3 ). Interviewed HCPs expressed challenges justifying a recommendation for ICSI to patients when semen analysis results are close to the threshold for inhibition of semen function:

*In 2010, WHO determined new standard parameters, e.g., 15 million sperms per mL, the mobility should be 32%, and at least 4% should be perfectly shaped.* […] *the threshold is so low that you say that everybody who gets below that, even in just one parameter, has a severe inhibition, thus an ICSI indication* […] *It's really difficult to understand. It's difficult to explain this to the couple.*

*–* Physician (Reproductive Medicine), Germany

Uncertainty about the eligibility criteria for an ART-related procedure was further confirmed by survey responses showing that 35% (113/323) of HCPs selected "ICSI" as opposed to "full andrological evaluation by a specialist" as the next best course of action for a case of a 45-year-old male and a 32-year-old woman who had had a previous IVF cycle resulting in 20 mature oocytes but poor embryonic development ( Appendix-4 ).

### 3) Challenges Maintaining Access to High-QualityFertility Care During a Pandemic

**Figure 2:**
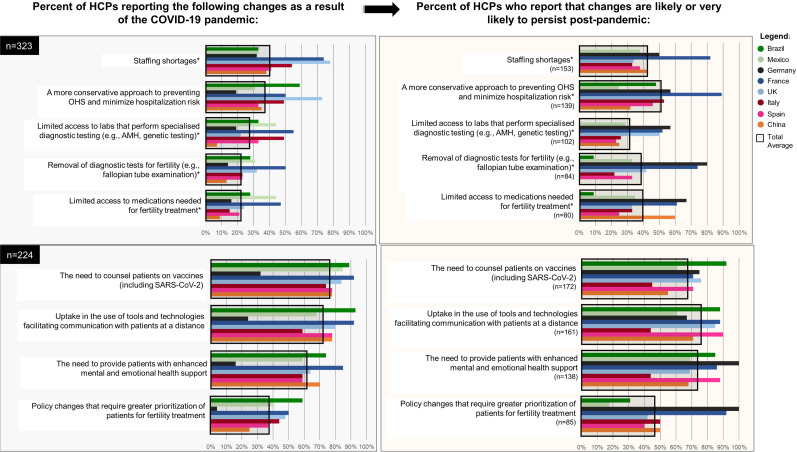
Percent of HCPs reporting changes as a result of the COVID-19 pandemic, and those likely to persist post-pandemic.

Enduring system-level changes resulting from the COVID-19 pandemic were reported by participants. Surveyed HCPs reported staffing shortages (47%, 153/323), limited access to laboratories for specialised diagnostic testing (32%, 102/323), cancellation of certain diagnostic tests (e.g., fallopian tube examination) from standard clinic procedures (26%, 84/323), and the introduction of changes to patient triaging policies (38%, 85/224). These challenges are illustrated by country in Figure-2 . Interviewed HCPs indicated that these institutional and organizational-related changes limited capacity to investigate and care for couples’ infertility:

*At a certain point, we had no time, because of all the extra bureaucratic work we had to do,* [including] *COVID procedures. In the past, we would execute tests to evaluate tubal functions,* [but] *at a certain point, we had to eliminate such testing. I think we haven't reintroduced them yet.*

– Physician (OBGYN), Italy

*The real impact was the prioritisation of patients. This is what had an impact on our activity. We were forced to adopt this strategy where we had to rank the degree of urgency of patients… When it comes to the protocol itself, it did not change. It is only the ranking that changed and giving priority to certain patients is something that is really difficult to do.*

– Laboratory Specialist, France

Additional expected professional competencies that emerged during the COVID-19 pandemic and expected to remain post-pandemic ( Figure-2 ), especially amongst physicians, included: the ability to counsel patients regarding the impact of SARS-CoV-2 vs. vaccines on fertility (77%, 172/224), increased use of telemedicine (72%, 161/224), enhanced provision of mental health and emotional support (62%, 138/224). Over 40% of surveyed HCPs (n=307) in all countries, except for France (31%, 8/26), rated their knowledge of the effectiveness of vaccines available to prevent SARS-CoV-2 infections and limit symptom severity at suboptimal levels. Over 40% of physicians in China, Spain, Italy, Mexico, and the UK reported suboptimal skill levels when discussing evidence of the risks versus benefits of SARS-CoV-2 vaccines on pregnancy and birth outcomes with patients ( Table-2 ). Despite using telemedicine and recognising the need for patient support, participants in China (48%, 19/40), France (32%, 8/25), Italy (44%, 11/25), and Mexico (56%, 14/25) reported suboptimal skill levels when building rapport with patients via telehealth during initial evaluations.

## DISCUSSION

While the COVID-19 pandemic has officially ceased in May 2023, the repercussions of this event are likely to persist and warrant a call for timely and tailored support for HCPs aiming to meet the continued and complex needs of patients moving along their reproductive journey. This study identified three challenge areas with underlying educational gaps (i.e., gaps in knowledge and skills) and barriers (i.e., beliefs or institutional/organizational level factors acting as hindrances) that could strategically inform tailored CME and CPD activities supporting physicians and laboratory specialists involved in ART and male infertility, in relation to: 1) investigating male-related infertility; 2) deciding appropriate treatment for men and selective use of ART-related procedure; and 3) maintaining high-quality fertility care during a pandemic. Identified challenges were reported by a substantial proportion of HCPs (>30%), both surveyed and interviewed, and corroborated with the interviews of patients who sought fertility care before and during the pandemic. These findings also align with challenges identified in pre-pandemic research, which strengthens the relevancy of the findings identified by this study ( 5 – 8 , 14 – 18 ). Commonalities found across perspectives, phases, and published literature, support the argument that the challenges, barriers and educational gaps identified in this study are likely present in the broader population of fertility professionals and merit immediate action ( 28 , 29 ).

An important topic investigated in this study, beyond the impact of COVID-19 on access to fertility care, is the ongoing dismissal of male-related factors of infertility, that can lead to inappropriate selection and use of an ART-related procedure, such as ICSI ( 14 , 37 – 39 ). This concern has been previously raised through an analysis of registry data from 79 countries, which indicated a steady increase in the number of ART-related procedures delivered globally over the last two decades, most of which have been ICSI-induced ( 40 ). This study provides evidence that physicians may recommend ICSI before rigorously investigating male's potential contribution to a couple's infertility. The following risk factors and indicators of male infertility can be missed by physicians: visual disturbances, lubricant use during sexual intercourse, and bariatric surgery ( 41 ). This study indicates a need to enhance HCPs’ knowledge of male contribution to couples’ infertility, including possible aetiologies of male infertility requiring thorough investigation, and eligibility criteria for a couple to be recommended an ART-related procedure like ICSI.

The overall suboptimal investigation of male infertility in clinical practice is a priority gap, given it can further complicate the treatment and management of couples aiming to conceive, especially within a narrow period window. A recent study found that couples tend to have lower quality of life when a male-related factor is at cause compared to exclusively female-related infertility ( 42 ). While a literature review conducted by Barratt, Björndahl ( 43 ) describes gaps in male infertility research that are yet to be bridged, the current treatment landscape allows HCPs to choose from a variety of treatments relevant to males that can truly optimise chances of conception, including but not limited to varicocele repair, sperm retrieval, transurethral resection of ejaculatory ducts, microsurgical reconstruction of genital tract, and medical treatments with selective oestrogen receptor modulators, antioxidants and gonadotropins ( 37 , 44 – 47 ). This study found that beyond the societal tendency to minimize male contribution to couples’ infertility, 10-30% of reproductive urologists and andrologists perceived their knowledge of currently available treatments for males to be less than advanced. These rates were even higher (30-70%) for other specialties (i.e., endocrinology, OBGYN), which tend to focus more on female infertility, but are still expected to demonstrate advanced knowledge in this domain to best serve couples in their reproductive journey. This may explain why almost 20% of physicians involved in ART and male infertility equally report skill gaps for setting realistic expectations with men about their fertility, and chances for conception ( 48 ).

In the context of ongoing transmissible viral infections and possible pandemics in the future, which can affect reproductive health, this study found that fertility care professionals perceive counselling patients on vaccines and using telehealth as both relevant skills to the optimal care of patients post COVID-19. This finding supports current efforts to better integrate immunology expertise and research into fertility care, as pregnancy is tied to unique regulatory changes in immunity ( 49 ). The skill of counselling patients on the efficacy of vaccines that physicians involved in fertility care report being relevant to their current practice would be most sensible with the support of an enhanced collaboration effort with immunology experts who can best inform patients about the potential risks and benefits of various vaccines on pregnancy and birth outcomes. This type of collaboration could help address patient concerns and health considerations in relation to the use of vaccines during pregnancy, including most recent advances against the SARS-CoV-2 virus ( 50 ), which continues to be a relevant health intervention today. In parallel, the introduction of telehealth consultation in fertility clinics appears to have been accelerated by the pandemic, given it offered a solution to stringent physical distancing measures. As found by this study, telemedicine is likely to persist post-pandemic internationally, given it can also be a useful tool to streamline patient monitoring and management via a facilitated access to healthcare professionals ( 51 , 52 ). However, a meaningful proportion of HCPs who participated in this study perceived their skills as less than advanced for building trust and empathy with patients via telemedicine. Assisting fertility care providers in developing relevant patient-facing skills using virtual communication platforms would be an important next step to ensuring that telehealth continues to be optimally integrated and used as a complement to in-person consultations ( 53 ). Studies on this topic are beginning to emerge, and highlight the opportunity to ensure telehealth consultations are carried out in a patient-centric way ( 54 ).

### Limitations

Despite a total sample size of 76 interview participants and 303 survey participants, given multiple countries were included in this study, the final sample size for the survey did not provide sufficient statistical power to assess significant differences in the identified gaps by country. Hence, only descriptive comparisons could be made. While chi-square analysis assessing variations by regions was possible, a larger representation of countries in each region would have increased the validity of results. The methodology selected for this study was mostly based on self-report. The limitation of self-reporting and recall bias was minimized by a triangulation of sources (i.e., patients, physicians and laboratory specialists) and methods (qualitative and quantitative findings), informed by a literature review at onset of the study ( 26 ).

## CONCLUSIONS

More efforts need to be placed in ensuring males’ aetiologies of infertility are thoroughly investigated in clinical practice and used to inform the appropriate use of ART in a couple's reproductive journey. These challenge areas are priority needs from the perspective of male and female patients, and tailored efforts in CME and CPD for reproductive medicine specialist should be deployed to support physicians and laboratory specialists involved in ART to acquire advanced levels of knowledge and skills to optimally address male infertility factors in a couple's reproductive journey. With the widespread integration of telemedicine in fertility care and efforts to promote healthy immunity and reproduction in the population, fertility care providers perceive a need to enhance their skills in effectively counselling patients on vaccines and/or using virtual platforms. Since the present study identified barriers at the health-system level, hindering the application of relevant knowledge and skills by HCPs, there is an additional need to better understand the required changes in policies and organizational processes that would facilitate access to andrology services for male infertility and specialized care, as needed.

## Data Availability

Aggregated and anonymized data will be made available for review or query upon reasonable request.

## APPENDIX

**Appendix 1:** Areas of explorations in Phase 1

The following table presents areas of potential challenges and barriers that were explored as part of interviews.

Areas of explorations	PR	LS	Px
Screening & diagnosis of infertility in both men and women	✓	✓	
Integration and use of different genetic testing & screening modalities	✓	✓	✓
Infertility treatment & procedures used for men versus women (e.g., gonadotropins, gamete assessment, selection & retrieval, ovarian stimulation, fertilization, embryo grading/selection, cryopreservation)	✓	✓	✓
Factors influencing treatment selection (including resources, guidelines and patient preferences)	✓		✓
Goals setting, expectations management and assessment/management of patients (both men & women) psychological needs	✓	✓	✓
Monitoring & encouraging treatment adherence (including gonadotropins) via various modalities (e.g., telehealth versus in-person)	✓	✓	✓
Patient-provider communication (including challenging topics of discussion, approach to discussing diagnosis & treatment, emotional support provided)	✓	✓	✓
Telehealth experiences (including factors considered in the selection of patients that would benefit from a telehealth consultations)	✓		✓
Impact of the COVID-19 pandemic on care provided to patient with fertility issues & approaches to support patients in coping with the pandemic	✓	✓	✓
Impact of the COVID-19 pandemic on the diagnosis, treatment & management of patients with fertility issues	✓	✓	✓
Impact of the COVID-19 pandemic on the organization of fertility services & interprofessional work	✓	✓	
Changes brought on by the COVID-19 pandemic expected to remain	✓	✓	✓

**PR** = physician in reproductive medicine; **LS** = laboratory specialist; **PX** = patient

**Appendix 2:** – Survey questions, items, responses and recoding for analysis.

Type of gap	Question/item	Responses	Recoding for analysis
Knowledge	To what approximate extent do males contribute to reported cases of infertility globally?	a. Less than 10% b. 10% - 30% c. 31% - 50% d. 50% or more	a or b = Gap c or d = No gap
	For each item listed below, please rate your current level of knowledge based on what is expected of your professional role in the care of patients seeking fertility care. Note: If item is NOT relevant to your current professional role, please select Not relevant ("NR").	NA	NA
	/Eligibility for in-vitro fertilization (IVF) and intracytoplasmic sperm injection (ICSI)	1=No knowledge at all; 2=Basic knowledge; 3=Intermediate knowledge; 4=Advanced knowledge; 5=Expert knowledge NR=Not relevant	1-3 = Gap 4-5 = No gap NR = Excluded from analysis
	/Microsurgical reconstruction of the male genital tract (e.g., vasovasostomy and vasoepididymostomy)		
	/Transurethral resection of ejaculatory ducts		
	/Techniques for varicocele repair		
	/Empirical medical treatment with selective estrogen receptor modulators, antioxidants, and gonadotropin in males		
	/Gonadotropin therapy for hormonal disorders in males		
	/Treatments for male patients that can *improve in vitro* fertilization (IVF) and intracytoplasmic sperm injection (ICSI) outcomes		
	/The effectiveness of vaccines available in preventing SARS-CoV-2 infections and limiting symptom severity		
Knowledge	Please rate your level of agreement with each of the following statements. Note: If you are unsure and/or not informed enough to form an opinion, please select "Unsure".	NA	NA
	/Poor embryonic development in an IVF cycle is an indication for ICSI.	1=Strongly disagree; 2=Disagree; 3=Unsure - not informed enough to form an opinion; 4=Agree; 5=Strongly agree	3-5 = Gap 1-2 = No gap
	/With the exception of 0% values (globozoospermia) even very low rates of sperm morphology have poor predictive power.		3-5 = Gap 1-2 = No gap
Skill	For each item listed below, please rate your current level of skill according to what is expected of your professional role in the care of patients seeking fertility care. Note: If item is NOT relevant to your current professional role, please select Not relevant ("NR").	1=No skill at all; 2=Basic skill level; 3=Intermediate skill level; 4=Advanced skill level; 5=Expert skill level NR=Not relevant	1-3 = Gap 4-5 = No gap NR = Excluded from analysis
	/Investigating hypothalamic-pituitary axis dysfunction in males		
	/Investigating spermatogenic defects		
	/Investigating ductal obstruction or dysfunction in males		
	/Investigating infectious disease causes of infertility in males		
	/Investigating systemic causes of infertility in males		
	/Counselling patients on the safety and efficacy of available vaccines (including ones for SARS-CoV-2)		
	/Discussing with patients’ evidence regarding the risks versus benefits of SARS-CoV-2 vaccines on pregnancy and birth outcomes		
	/Building rapport with patients via telehealth during an initial evaluation		
Skill (decision-making)	A 45-year-old man and a 32-year-old woman present because they experienced an unsuccessful IVF cycle in another clinic 4 months prior. The previous IVF attempt resulted in 20 mature oocytes and poor embryo development.	NA	NA
	/Which assessments would be obtained to guide further treatment? Check all that apply.	a. Anti-Müllerian hormone b. Anti-sperm antibody titer c. Semen analysis/sperm function test d. Serum testosterone for male e. Serum FSH for male and female f. Genetic tests	a, b, d, e or f = Incorrect c = Correct
	/All results are normal except semen analysis/sperm function test - 3% of sperm with normal morphology. What is the next best course of action?	a. A second cycle of conventional IVF b. Split-cycle IVF/ICSI c. ICSI d. Full andrological evaluation by a specialist	a-c = Incorrect d= Correct
Attitude	Please rate your level of agreement with each of the following statements. Note: If you are unsure and/or not informed enough to form an opinion, please select "Unsure".	NA	NA
	/Investigating the causes of male infertility is simpler than for female infertility.	1=Strongly disagree; 2=Disagree; 3=Unsure - not informed enough to form an opinion; 4=Agree; 5=Strongly agree	4-5 = Gap 1-3 = No gap
Performance	How often do you consider the health of a female patients’ male partner when investigating and treating their fertility issues?	a. Always b. Most of the time c. Sometimes d. Rarely e. Never	b-e = Gap a = No gap
System	Has the COVID-19 pandemic resulted in the following changes? If so, what is the likelihood of each change persisting after the COVID-19 pandemic is over?	NA	NA
	/staffing shortages	Part 1. a. Yes b. No Part 2. 1=Very unlikely 2= Unlikely 3= Unsure 4 = Likely 5 = Very likely	Part 1. a = Change b = No change Part 2. 4=5 = Likely or very likely 3 = Unsure 1-2 = Unlikely to very unlikely
	/removal of diagnostic tests for fertility (e.g., fallopian tube examination)		
	/limited access to labs that perform specialised diagnostic testing (e.g., Anti-Müllerian hormone, genetic testing)		
	/limited access to medications needed for fertility treatment		
	/policy changes that required a greater prioritisation of patients for fertility treatment than before		
	/a more conservative approach to preventing ovarian hyperstimulation syndrome and minimise hospitalisation risk		
	/the need to counsel patients on vaccines (including SARS-CoV-2)		
	/an uptake in the use of tools and technologies facilitating communication with patients at a distance		

**Appendix 3:** – Percentage of HCPs agreeing, unsure or disagreeing with opinion statements.

Statement	Answer	Countries (%, n)								
		South America		Western Europe			Southern Europe		Asia	Mean Total
		BR	MX	DE	FR	UK	IT	SP	CH	Total
Investigating male infertility is simpler than female infertility *	Disagree	54% (21/39)	33% (13/39)	3% (1/37)	37% (14/38)	46% (17/37)	49% (19/39)	44% (17/39)	31% (17/55)	37% (119/323)
	Unsure	0% (0/39)	21% (8/39)	32% (12/37)	29% (11/38)	16% (6/37)	28% (11/39)	21% (8/39)	18% (10/55)	20% (66/323)
	Agree	46% (18/39)	46% (18/39)	65% (24/37)	34% (13/38)	38% (14/37)	23% (9/39)	36% (14/39)	51% (28/55)	43% (139/323)
With the exception of globozoospermia, even very low rates of sperm morphology have poor predictive power *	Disagree	33% (13/39)	21% (8/39)	8% (3/37)	3% (1/38)	22% (8/37)	15% (6/39)	8% (3/39)	11% (6/55)	15% (48/323)
	Unsure	26% (10/39)	33% (13/39)	49% (18/37)	24% (9/38)	24% (9/37)	36% (14/39)	36% (14/39)	22% (12/55)	31% (99/323)
	Agree	41% (16/39)	46% (18/39)	43% (16/37)	74% (28/38)	54% (20/37)	49% (19/39)	56% (22/39)	67% (37/55)	55% (176/323)
Poor embryonic development in an IVF cycle is an indication for ICSI	Disagree	15% (6/39)	18% (7/39)	5% (2/37)	11% (4/38)	22% (8/37)	13% (5/39)	23% (9/39)	13% (7/55)	15% (48/323)
	Unsure	3% (1/39)	26% (10/39)	30% (11/37)	16% (6/38)	35% (13/37)	18% (7/39)	5% (2/39)	31% (17/55)	21% (67/323)
	Agree	82% (32/39)	56% (22/39)	65% (24/37)	74% (28/38)	43% (16/37)	69% (27/39)	72% (28/39)	56% (31/55)	64% (208/323)

BR (Brazil); CH (China); FR (France); DE (Germany); IT (Italy); MX (Mexico); SP (Spain); UK (United Kingdom).

Five-point agreement rating scale was used (1=Strongly disagree, 2=Disagree, 3=Unsure – Not informed enough to form an opinion, 4=Agree, 5=Strongly agree).

* Asymptotic significance (2-sided) < 0.05 for 3x4 (agreement X region) crosstabulation with Pearson Chi-Square statistical test

**Appendix 4:** HCP survey responses to a clinical case question.

Question item	Answer	South America		Western Europe			Southern Europe		Asia	Total
		BR	MX	DE	FR	UK	IT	SP	CH	
Which assessments would be obtained to guide further treatment? Check all that apply.	Anti-Müllerian hormone	15% (6/39)	31% (12/39)	70% (26/37)	37% (14/38)	35% (13/37)	28% (11/39)	36% (14/39)	53% (29/55)	39% (125/323)
	Anti-sperm antibody titer	26% (10/39)	28% (11/39)	73% (27/37)	26% (10/38)	41% (15/37)	54% (21/39)	26% (10/39)	76% (42/55)	45% (146/323)
	**Semen analysis/sperm function test** *	**82%** **(32/39)**	**82%** **(32/39)**	**89%** **(33/37)**	**95%** **(36/38)**	**76%** **(28/37)**	**77%** **(30/39)**	**74%** **(29/39)**	**91%** **(50/55)**	**84%** **(270/323)**
	Serum testosterone for male	26% (10/39)	64% (25/39)	76% (28/37)	24% (9/38)	30% (11/37)	41% (16/39)	28% (11/39)	58% (32/55)	44% (142/323)
	Serum FSH for male and female	21% (8/39)	74% (29/39)	73% (27/37)	45% (17/38)	30% (11/37)	54% (21/39)	33% (13/39)	73% (40/55)	51% (166/323)
	Genetic tests	79% (31/39)	46% (18/39)	35% (13/37)	34% (13/38)	46% (17/37)	38% (15/39)	67% (26/39)	85% (47/55)	56% (180/323)
All results are normal except semen analysis/sperm function test - 3% of sperm with normal morphology. What is the next best course of action?	A second cycle of Conventional IVF	5% (2/39)	13% (5/39)	35% (13/37)	3% (1/38)	8% (3/37)	15% (6/39)	5% (2/39)	7% (4/55)	11% (36/323)
	Split-cycle IVF/ICSI	23% (9/39)	26% (10/39)	8% (3/37)	16% (6/38)	24% (9/37)	15% (6/39)	36% (14/39)	20% (11/55)	21% (68/323)
	ICSI	41% (16/39)	31% (12/39)	41% (15/37)	26% (10/38)	16% (6/37)	26% (10/39)	41% (16/39)	31% (17/55)	35% (113/323)
	**Full andrological evaluation by a specialist** *	**31%** **(12/39)**	**26%** **(10/39)**	**14%** **(5/37)**	**53%** **(20/38)**	**16%** **(6/37)**	**44%** **(17/39)**	**15%** **(6/39)**	**40%** **(22/55)**	**30%** **(98/323)**
	I do not know	0% (0/39)	5% (2/39)	3% (1/37)	3% (1/38)	5% (2/37)	0% (0/39)	3% (1/39)	2% (1/55)	2% (8/323)

Case: A 45-year-old man and a 32-year-old woman present because they experienced an unsuccessful IVF cycle in another clinic 4 months prior. The previous IVF attempt resulted in 20 mature oocytes and poor embryo development.

* **Correct answer**
